# The Prevalence of Smoking (Cigarette and Waterpipe) among University Students in Some Arab Countries: A Systematic Review

**DOI:** 10.31557/APJCP.2020.21.3.583

**Published:** 2020-03

**Authors:** Abdulsalam M A Nasser, Yarui Geng, Samer Abdo Al-Wesabi

**Affiliations:** 1 *School of Medicine and Health Management, Tongji Medical College, Huazhong University of Science and Technology, *; 2 *Wuhan Aige Ophthalmic Hospitals. No: S-8 Building, Nande International Area, Qian Chuan Street, Huangpi, Hubei Province, Wuhan, China. *

**Keywords:** Prevalence, cigarette and waterpipe, students, Arab countries, systematic review

## Abstract

**Background:**

Tobacco use among university students remains the most alarming problem worldwide. This study aims to systematically review the previous literature for determining the prevalence of smoking (cigarette and waterpipe) among university students in some Arab countries.

**Methods:**

We electronically searched articles from MEDLINE, PubMed, EMBASE, Google Scholar and Google for the period from April 2018 to June 2019. We conducted a systematic review of eligible studies published in English between 2006 and 2019, for assessing cigarette and waterpipe smoking among university students. The studies were all cross-sectional according to eligibility criteria and contained 469 studies19 meet the inclusion criteria from 12 countries of (Yemen, Kingdom of Saudi Arabia (KSA), Kuwait, Bahrain, Jordan, Lebanon, Tunisia, Egypt, Palestine, Syria, Libya and United Arab Emirates (UAE).

**Results:**

the study included a total of participants (N=45,306) (33,450 Males vs 11,856 Females). The overall highest rate of current smoking among students was in Egypt (46.7%), Kuwait (46%) and KSA (42.3%). The smoking prevalence among males was significantly higher than females in Yemen (36.3% vs 28.0%,p<0.001), Bahrain (27.0%vs 4.2%, p<0.001), Tunisia (38.4% vs 3.4%, P<0.001), Egypt (61.2% vs 18.9%, P<0.001), Palestine (52.7% vs 16.5%, p<0.001), Syria (26.1% vs 9.5%, p<0.001), KSA (32.7%vs5.9%,P<0.001), and Jordan (54.3%vs11.1%, P<0.005) and (56.9%vs11.4%, P<0.005). Another study in Yemen was significantly higher among women than man (15.7% vs 10.3%, p<0.001). The highest waterpipe smoking rates among gender was in KSA (36.4%-36.3%). For cigarette smoking, the highest rates were in Libya (80.2%), Jordan (80%) and KSA (70.7%). The highest smoking rates among males were in Egypt (61.2%), Jordan (56.9%-54.3%) and Palestine (52.7%), for females the highest rate was in Yemen (28.0%).

**Conclusion:**

The prevalence of smoking cigarette and waterpipe appears to be alarmingly high among university students in Arab countries. The results were different among students, due to the nature of the region and the different customs, traditions, lineage and multicultural from country to another.

## Introduction

Tobacco smoking is a leading global disease risk factor, ill health, and deaths (Drope and Schluger, 2018). It is projected that, if the trend of tobacco consumption persists, 8 million people will die yearly by 2030 (CDCP, 2019). According to the World Health Organization (WHO), it’s estimated that tobacco is the reason for more than 7 million deaths and hundreds of billions of dollars loss, worldwide each year (WHO, 2017). Deaths due to tobacco smoking are not only preventable tragedies but have an important economic cost. Worldwide, the total economic damage of smoking has been estimated at more than United State (US)$ 1.4 trillion per year (Goodchild et al., 2018) equivalent to 1.8 percent of the world’s annual Gross Domestic Product (GDP). Although tobacco smoking has declined in many developed countries, 80% of the 1.1 billion smokers who live in low-and-middle income countries continue to suffer the burden of tobacco-related consequences (Drope and Schluger, 2018).

Moreover, most of the world’s current smokers started the habit during their adolescence. In a similar study, the duration of smoking inversely associated with the age of initiation (Mackay et al., 2006). Globally, the number of cigarettes smokers’ adolescent, boys and girls, has been estimated to be as 25 million and 13 million (ACS, 2018). This habit has become a pandemic responsible for about 6 million deaths annually (WHO, 2018).

In the Eastern Mediterranean Region (EMR), tobacco prevalence is underestimated because of a number of reasons including lack of data from some countries, underreporting by females, and the presence of unaccounted methods of tobacco consumption (e.g. water-pipe) (Mandil et al., 2013). Moreover, a recent national survey showed that the overall prevalence rate of tobacco smoking was 12.2% for aged 15 years old or older with 4.3% of who smoke waterpipe every day and 1.4% of those who currently smoke cigarettes and waterpipe users (Moradi-Lakeh et al., 2013).

Smoking prevalence remains highly significant in most of the Arab countries (Yemen, Lebanon, Jordan, Egypt, Tunisia, Syria, and Iraq), they have very high adult male smoking prevalence rates, Yemen and Djibouti have some of the highest male smoking prevalence rates in the world, above 75%, rates of male smoking are also exceptionally high, above 40%, in Jordan, Tunisia, Egypt, Lebanon, Syria and Palestine, smoking prevalence among females in Arab countries is generally low, under 10%, except Egypt, Lebanon and Yemen, a larger percentage of females in Lebanon and Yemen smoke tobacco than females in the United States, there are more than 12 Arab countries were at least 10% of girls aged 13–15 smoke, this indicates a dangerous trend toward more widespread women smoking in Arab world (Shafey, 2007 ; Jha et al., 2002). In addition, recent studies in some Arab countries, gave different rates of smoking among students, it still alarming rates, such as KSA among applied medical sciences college’s students 72% (AlQahtani, 2017), Yemen 33.1% (Nasser and Zhang, 2019), Egypt 46.7% (Khan et al., 2012), Kuwait 46% (Husain et al., 2016), Libya 28.3% (Abou-Faddan and Ahmed, 2012) and Lebanon 26% (Jradi et al., 2013).

As researchers, we were unable to reach a consensus about the actual prevalence of smoking among students in Arab countries. For that the present systematic review to examine and analyze data to estimate the pooled prevalence of smoking among students in some Arab countries.

The goal was to compare this study’s results with the level findings in some Arab countries. The purpose of these comparisons is to determine and knowledge the overall prevalence of smoking and its severity within the Arab countries and region. This study is aimed to inform decision makers in Arab countries, field health researchers, individuals and society about the smoking problem, so that they can design effective tobacco control interventions. We need to have multiple approaches to prevent and control the programs that target adults and teenagers. 

## Materials and Methods

*Methodology*

*Study Design*

We conducted a systematic review of the previous research articles, published in English, about cigarette and waterpipe use among universities students in some Arab countries in the period between 2006 and 2019. The current study included cross-sectional studies among smokers at Arab universities. The total participants were (n= 45,306). The study included 19 studies from 12 Arab countries; Yemen (Nasser and Zhang, 2019; Nasser et al., 2018), KSA (Al-Ghaneem et al., 2016; Hassan et al., 2014; Mandil et al., 2011; Mandil et al., 2010), Kuwait (Husain et al., 2016), Bahrain (Alzayani and Hamadeh, 2015), Jordan (Obeidat et al., 2014; Khader and Alsadi, 2008; Alomari et al., 2006), Lebanon (Jradi et al.,2013), Tunisia (Maatouk et al., 2013), Egypt (Khan et al., 2012; Abolfotouh et al., 2007), Palestine (Musmar, 2012), Syria (Al Kubaisy et al., 2012), Libya (Abou-Faddan and Ahmed, 2012) and UAE (Mandil et al., 2007) as shown in (Table1). 

We extracted the data from each study selected according to gender, age, cigarette and waterpipe smokers, study design, participants’ number, response rate of participants, publication year and authors of the study (according to systemic review study, the prevalence of tobacco use among students aged 15 to 37) (Table 1). The study included both sexes students from different colleges, health-related and non-health related colleges were selected (to ensure good representation of the sample). 

*Search Strategy*

We electronically searched articles from MEDLINE, PubMed, Google Scholar and Google to identify relevant research, for the period April 2018 to June 2019. The search string was: (Smoking OR Tobacco OR Tobacco smoking OR Tobacco prevalence OR Cigarette smoking OR waterpipe smoking OR Cigarette OR Cigar OR waterpipe). We chose studies using a phase duplicate and independent screening process. The study included cross-sectional studies to evaluate the prevalence of use of cigarette and waterpipe among the universities students in some Arab countries (Table1). We summarized the primary results of each study of data that appeared during the information extraction process.

*Data Extraction*

Data extraction (AMAN, YG and SAAW) three reviewers used a standardized and pilot tested form to collect data from each qualified study using a duplicate and autonomous screening process and then independently extracted data (i.e. smokers, gender, age, cigarette smoking, waterpipe smokers, study design, participants’ number, response rate of participants, publication year and authors of the study (according to systemic review study) from each article and evaluated them based on exclusion criteria and the Russell and Gregory guidelines (Russell et al., 2003).

*Selection Criterion*

In this study, the selection criterion had some inclusion criteria: a focus on university students in Arab countries, the topic of data collection was restricted to who smoked cigarettes and waterpipes, among students (aged 15-37) and we accepted other definitions in the research literature (eg, aged 16-32 years, 18-28 years and17-37 years).

**Figure 1:**
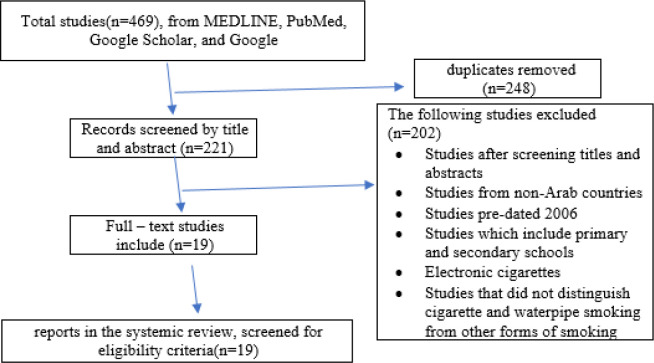
Flow Diagram of Studies in this Systematic Review

We applied this definition to the age group between 15 years and 37 years. The researchers excluded other forms of tobacco, such as electronic cigarettes and others because of their lack of existing research in their field, studies that did not distinguish cigarette and waterpipe smoking from other forms of smoking, studies reported as abstracts and for which we could not identify as a full text, studies from non-Arab countries, studies which include primary and secondary schools, and studies pre-dated 2006 (Figure 1). For the convenience of reporting the findings, the researchers agreed to unify the names of similar medical colleges or health-related under one name, as we did in other related non-health colleges. Current smokers were defined as university students who had smoked at least once or twice within the previous 30 days, (Maziak et al., 2005; WHO, 1998). The subject of the investigation was restricted to universities students who smoked cigarettes and waterpipe; the researchers excluded other forms of tobacco

*Statistical Analysis*

We analyzed compare by country, age group and prevalence of smoking (CIGARETTE and WATERPIPE) among students. We used a standardized trend to collect data from each qualified study using a duplicate and independent screening process. A total of nineteen studies that met the inclusion criteria were identified and analyzed.

## Results

*Description of Included Studies*

Table 1 shows the distribution of the included studies (1; smokers, 2; gender, 3; age, 4; cigarette smokers, 5; waterpipe smokers, 6; study design, 7; participants’ number, 8; response rate of participants, 9; publication year and 10; authors of the study). Figure1 shows the 469 articles from MEDLINE, PubMed, EMBASE, Google Scholar, and Google: We excluded the following: 202 studies after screening titles and abstracts, studies from Non-Arab countries, studies pre-dated 2006, studies that did not distinguish cigarette and waterpipe from other forms of smoking, studies which include primary and secondary schools, electronic cigarettes and 248 duplicates removed. Nineteen reports in this review screened for quality and eligibility criteria.

*Assessment of Methodology of the Studies*

All studies were cross-sectional and these studies intended to document the prevalence (cigarette and waterpipe smoking) and to determine and knowledge the prevalence of smoking and its severity within the Arab countries. The sample selection process was described clearly in all studies and the prevalence rates and characteristics of samples were reported in most studies. All these papers used criterion tools /instruments.

*Instruments Used*

In this literature review, a variety of tools were used to evaluate the prevalence of smoking among university students. The different instruments used were: Questionnaires and data collection tool gathered from 19 studies in these countries: [Yemen (Nasser and Zhang, 2019; Nasser et al., 2018) using the Global Youth Tobacco Survey (GYTS) and Global Health Professional Survey (GHPS) (WHO and GYTS, 1998; WHO and GHPS, 2004). [KSA (Al-Ghaneem and Al-Nefisah, 2016) using a self-validated and pre-tested questionnaire, KSA (Hassan et al., 2014) using modified Arabic version of the (GYTS) questionnaire (GYTS,1998), KSA (Mandil et al., 2011) using a modified version of the (GYTS/CDCP core questionnaire) (GYTS, 2008), and KSA (Mandil et al., 2010) using a modified version of the WHO/CDC/GYTS (GYTS, 1998) was used for data collection]. [Kuwait (Husain et al., 2016) using a pretested questionnaire for details on demographics and health complaints]. [Bahrain (Alzayani and Hamadeh, 2015) using a self-administered anonymous questionnaire, and abridged from the adult question sheet of the (UAE) Health and Lifestyle Survey (Badrinath et al., 2002)]. [Jordan (Obeidat et al., 2014) using a self-administered questionnaire to a convenience sample of students, Jordan (Khader and Alsadi, 2008) using a pilot-tested structured questionnaire, prepared specifically for the study, and Jordan (Alomari et al., 2006) from Hiroshima University-Dental Behavior Inventory (HU-DBI) survey (Kawamura et al., 2000) was used a modified English version]. [Lebanon (Jradi et al., 2013) using a self-administered based on the GHPS/CDCP (GHPS and CDCP, 2008), questions developed by Maziak et al, and also contained the items which developed (Maziak et al., 2005; Almerie et al., 2008)]. [Tunisia (Maatouk et al., 2013) using a survey using a self-administered questionnaire]. [Egypt (Khan et al., 2012) using a modified (GHPS) revised in January 2007 as part of the GTSS (Warren, 2006; Warren, 2009; Warren et al., 2008), and Egypt (Abolfotouh et al., 2007) using by a pre-designed self-reported questionnaire]. [Palestine (Musmar, 2012) using a questionnaire adopted from the GYTS and the GHPS (WHO and GYTS, 1998; WHO and GHPS,2004)]. [Syria (Al Kubaisy et al.,2012) using a pre-tested questionnaire was designed based on those used in similar surveys (Kin, 2009; Almerie et al., 2008)]. [Libya (Abou-Faddan and Ahmed, 2012) using an anonymous self-administered questionnaire] and [UAE (Mandil A et al.,2007) using question sheet a modified version of the WHO and the GYTS (WHO,1983; GYTS, 2012), it was developed by researchers as an anonymous self-administered questionnaire].

*Characteristics of the Studies*

All of the included studies were cross-sectional descriptive studies within universities in some Arab countries. The overall sample size of all included studies was 45,306 participants: 33,450 males and 11,856 females. The highest response rate among males’ students was in KSA (99%), the highest response among female rate was in Libya (95%) and lowest response rate was in Lebanon among both sexes (54.3%) respectively (Al-Ghaneem and Al-Nefisah, 2016; Abou-Faddan and Ahmed, 2012; Jradi et al., 2013). Table 1 shows the all studies collected with overall smoking prevalence size from KSA included four studies (42.3% - 30.4% -14.5% and 14.5%) (Hassan et al.,2014; Al-Ghaneem and Al-Nefisah, 2016; Mandil et al., 2011, Mandil et al., 2010), Jordan three studies (35.0%-23.0% and 17.2%) (Khader and Alsadi,2008; Obeidat et al., 2014; Alomari et al., 2006) , Yemen two studies (33.1%-12.4%) (Nasser and Zhang, 2019; Nasser et al., 2018), and Egypt two studies (46.7%-7.7%) respectively (Khan et al., 2012; Abolfotouh et al., 2007). Some other countries included only one study as in Palestine (34.7%) (Musmar, 2012), Libya (28.3%) (Abou-Faddan and Ahmed , 2012), Lebanon (26.3%) (Jradi et al., 2013), Syria (20.8%) (Al Kubaisy et al., 2012), UAE (15.1%) (Mandil et al., 2007), Tunisia (14%) (Maatouk et al., 2013), Kuwait (46%) (Husain et al., 2016) and Bahrain (10.8%) (Alzayani and Hamadeh, 2015).

*Prevalence of Smoking among Students (Both Sexes) According to Ages*

Nine of 19 studies show that prevalence of smoking among male students were significantly higher than females in Yemen (36.3% vs 28.0%, p<0.001) for age18-24 years (Nasser and Zhang, 2019), Bahrain (27.0% vs 4.2%, p<0.001) for age 19 ≥22 years (Alzayani and Hamadeh, 2015), Tunisia (38.4% vs 3.4%, P<0.001) for age 18-28 years (Maatouk et al., 2013), Egypt (61.2%vs18.9%, P<0.001) for age20-27 years (Khan et al., 2012), Palestine (52.7%-16.5%, p<0.001) for age 18-20 years (Musmar, 2012), Syria (26.1% -9.5%, p<0.001) for age 17-28 years (Al Kubaisy et al., 2012), and KSA (32.7% vs 5.9%, P<0.001) for age 17-25 years (Mandil et al., 2011). In Jordan, the smoking predictors were significant (54.3% vs 11.1%, P<0.005) and (56.9% vs 11.4%, P<0.005) for age 19 ≥ 22 years and 17-28 years (Obeidat et al., 2014; Khader and Alsadi, 2008) respectively. In Yemen, another study was significantly higher among females than males (15.7% vs10.3%, p<0.001) for age18-24 years (Nasser et al., 2018). We found 4 from 19 studies were higher among males than females, but not significant in KSA (Mandil et al., 2010), Egypt (Abolfotouh et al., 2007), Jordan (Alomari et al.,2006) and UAE (Mandil et al., 2007) (Table 1).

Four studies did not mention the prevalence rate among both sexes but we included them. Three studies indicate that prevalence among males in KSA (30.4% - 42.3%) for age 18-25 years and 18-22 years (Al-Ghaneem and Al-Nefisah, 2016; Hassan et al., 2014), and Kuwait (46%) for age 16-32 years (Husain et al., 2016). One study in Libya among female was (28.3%) for age19-25 years (Abou-Faddan and Ahmed, 2012). 

In 6 out of 19 studies, the highest rate for prevalence among males were in Egypt (61.2%) for age20-27 years (Khan et al., 2012), Jordan (56.9%-54.3%) for age 17-28 years and 19≥22 years (Khader and Alsadi, 2008; Obeidat et al., 2014), Palestine (52.7%) for age18-20 years (Musmar, 2012), Kuwait (46%) for age 16-32 years (Husain et al., 2016), KSA (42.3%) for age 18-22 years (Hassan et al., 2014) and Tunisia (38.4%) for age 18-28 years (Maatouk et al., 2013). And among females, the highest rate was in Yemen (28.0%) for age 18-24 years (Nasser and Zhang et al., 2019) (Table1).

*Prevalence of Smoking Cigarette and Waterpipe*

Fourteen from 19 studies provided different results, five studies were the highest rate prevalence of cigarette smoking in Libya (80.2%) (Abou-Faddan and Ahmed, 2012), Jordan (80%) (Khader and Alsadi, 2008) and KSA (70.7%-48.2%-48.1%) respectively (Al-Ghaneem and Al-Nefisah, 2016; Mandil et al., 2011; Mandil et al., 2010), and 3 studies were the lowest rate in Jordan (3.5%) (Obeidat et al., 2014), Yemen (7.4%) (Nasser et al., 2018) and UAE (9.4%) (Mandil et al., 2007). 

Regarding waterpipe smoking, three studies were the highest rate prevalence in KSA (36.4%-36.3%) (Mandil et al., 2011; Mandil et al., 2010), and Lebanon (29.5%) (Jradi et al., 2013), and 3 studies were the lowest rate in Bahrain (2.0%) (Alzayani et al., 2015), Yemen (5.0%) (Nasser et al., 2018) and UAE (5.6%) (Mandil et al., 2007) (Table1).

## Discussion

Related to the number of studies on smoking, a few studies in universities focused on the cigarette and waterpipe smoking among both sexes of students. Due to the different19 studies, we reviewed it was difficult to find direct comparisons among the studies. Despite these challenges, this systematic review provides information on what is known about students who smoke cigarettes and waterpipe with different age groups in Arab countries. In this systematic review of more than 45,306 participants and 19 studies from 12 countries, we found a high prevalence of smoking cigarette and waterpipe among students in some Arab countries for age ≥ 15 to 37years. And the results indicated the prevalence of youth smoking varied widely among Arab students. Overall, the highest percentage of tobacco smokers was 46.7%, 46%, and 42.3% in Egypt, Kuwait and KSA respectively (Khan et al., 2012; Husain et al., 2016; Hassan et al., 2014). These results are in agreement with the report by the Middle East and North Africa (MNA) which indicated the prevalence smoking of youth varied widely in Arab area and associated with bad health outcomes (ET/MENA, 2001).

This review shows that the prevalence rate of smoking in Yemen, Palestine, Bahrain, Jordan, Tunisia, Egypt, UAE and KSA had a higher prevalence which were observed among males more than females (36.3% vs 28.0%), (52.7% vs 16.5%), (27.0% vs 4.2%), (54.3% vs 11.1% and 56.9% vs 11.4%),(38.4% vs 3.4%), (61.2% vs 18.9%), (33.0% vs 3.9%) and (32.7% vs5.9%) respectively (Nasser and Zhang, 2019; Musmar, 2012; Alzayani and Hamadeh, 2015; Obeidat et al., 2014; Khader and Alsadi, 2008; Maatouk et al., 2013; Khan et al., 2012; Mandil et al., 2005) and (Mandil et al., 2011) (Table1). This is compatible with many studies conducted in Mediterranean and Arab countries that indicates a significantly higher prevalence of smoking among males, which may be due to the social acceptability of the smoking habit among males (Metintaş et al., 1998; Haddad and Malak, 2002; Hasim, 2000; Melani et al., 2000; Bener et al., 1999). A literature review of smoking in KSA (Bassiouny, 2009) confirms that smoking was reported to be higher among males compared to females, regardless of the age group of study (school children, university students, adults). This finding may be attributed to the lack of awareness or comprehension of smoking risks to health in addition to the sales promotion by tobacco commercials which increased pressure on young people due to problems area and poor economic situations in some Arab countries.

A recent study in YEMEN 2019, which is in this review, we observed a higher smoking prevalence among females more than males and it was statistically significant (21.1% vs 1.9%, p<0.001), and indicated an increased prevalence of waterpipe usage among females than males (Nasser and Zhang, 2019). The predominance of waterpipe smoking among females reflects the replacement of cigarette smoking by waterpipe smoking as a sign of prestige among young people in the Eastern Mediterranean Region (Chaaya et al., 2004); as review shown in UAE among females (26.2% waterpipe smokers versus 8.9% cigarette smokers) (Mandil et al., 2007). Moreover, waterpipe smoking is considered more acceptable than cigarette smoking for women (Maziak et al., 2004); this was unlike the study found in Egypt 2012 there was high rates waterpipe use among males than females (49.0% vs16.3%) (Khan et al., 2012), the reasons beyond this could be associated to people’s understanding toward smoking, as they think that it’s socially accepted and consider it to be less harmful than other forms of tobacco (Israel et al., 2003; WHO, 2006). Based on my personal observation, smoking prevalence has recently increased among females in Arab countries’, particularly waterpipe smoking.

The review the rate prevalence of waterpipe smoking was highly in KSA (36.4%-36.3%) (Mandil et al., 2011; Mandil et al., 2010), with percentage more prevalence than cigarette using in Lebanon (29.5% vs 26.3%), Egypt (17.6% vs 17.4%) and Jordan (12.6% vs 3.5%) respectively (Jradi et al., 2013; Khan et al., 2012; Obeidat et al., 2014). The findings related to the prevalence of waterpipe smoking in this review are similar to those of previous studies among students in rural areas of Yemen (Nasser et al., 2018). Similarly, surveys found alarming prevalence of current waterpipe smoking among adults was the following: Gulf region (4%-12%) (Taha, 2007; Milaat et al., 1999; Behbehani et al., 2004). Our results agree with national surveys conducted by the GYTS and the WHO in a number of nations (Portney and Mary, 2015). As noted in the literature, there are significant differences among students in the prevalence rate of smoking from one country to another. The differences in Arab countries may be due to differences in cultural and social habits that ascribe different meanings to being a smoker.

The majority of studies reported high prevalence smoking when the study population was small and specific, as KSA (42.3%) for males (Hassan et al., 2014), in Egypt (46.7%) for both sexes (Khan et al., 2012) and Libya (28.3%) for females (Abou-Faddana and Ahmed, 2012). Whereas the prevalence would be more representative when the population size is large and diverse. For instance, many studies addressed smoking in health-related colleges among students or in non-health-related colleges; this was not representative of the whole university population, and thus, most of them showed the highest prevalence of smoking. One explanation could be that the majority of health-related researchers preferred to conduct their research on convenient and approachable health-related students. This technique of sampling could create a potential bias of self-selection, where a student may be unduly influenced by motivation, interest, or health consciousness about the phenomenon (Portney and Mary, 2015).

As stated above, smoking of cigarette and waterpipe was consistently more common in universities students than in adults in countries in which studies were conducted. In the absence of longitudinal data, it is not clear whether these results reflect a time trend of age-specific prevalence. If the specific prevalence of smoking has been increasing with age, the observation suggests that students tend to smoke as they reach their adulthood and increases with years of study. Unlike the study from Tunisia in this review, was that the frequency of smoking did not seem to vary with age, except after 25 years (p<10-3), In addition, the progression of students in the studies did not seem to influence the prevalence of smoking (p>0.05) (Maatouk et al., 2013). another study from Jordan 2006, was obvious that prevalence decreased with progression of the students in their academic studies (33.3%) first-year students compared to (3.7%) in the fourth year. However, smoking prevalence increased again among final-year students. The reason for this is difficult to explain, maybe due to the different kinds of stresses which students are exposed to during their final year at the university (Alomari et al., 2006).

In systematic review, by recent study in Yemen, confirms that smoking rate increase with age and years of study, producing a significant association between student age and the tendency to smoke (p<0.001) (Nasser and Zhang, 2019), in Egypt, there was a high rate of smoking among final-year medical students is notable (Khan et al., 2012), in KSA smoking was significantly associated with age of students where the probability of smoking increased with older ages (Hassan et al., 2014), other study in KSA a highly statistically significant rising trend of smoking risk was observed from freshmen in their 1st year of study to final year students (P<0.001) (Mandil et al., 2011), in Palestine, it was higher among older students than younger ones (Musmar, 2012), in Jordan the prevalence of smoking increased significantly with higher number of years of university education (P<0.0001) (Khader and Alsadi, 2008), and as confirmed by other similar studies (Rachiotis et al., 2008; Sanchez et al., 2010). This may be due to the longer exposure of senior students to older smokers within the university environment (Teachers, Workers, Friends, etc.) who could strongly influence their attitudes. and also, maybe due to family pressure against youth smoking that lessens as individuals acquire more freedom. If age-specific prevalence has been growing, this indicates that students have been affected the most and we might see a group effect of increasing spread among adults as these younger individuals age. Thus, it was difficult to compare between reports because of the different ways of defining smoking, age groups, and different methodologies adopted. 

*Limitations*

Firstly, the various limitations in this study were the high number of instruments used, difficulty in data collection and spaced locations in studies, and the result of this review could not represent the smoking prevalence among students in Arab universities. However, this was an attempt to estimate and understand the pooled estimate of smoking prevalence among included studies in this review. Secondly, all the studies were cross-sectional which provided the measurement of a certain population of interest rather than examining any association or causation. As was noted, culture barriers play a crucial role in reporting the real prevalence. Thirdly, this study may have been influenced by the cultural and societal biases reported by some studies, which may have underestimated the actual pooled estimate among students’ participants.

*Conclusion and Recommendations*

The cigarette and waterpipe smoking are a health problem among university students in Arab countries. such as (Egypt, Kuwait, KSA, Jordan, Libya, Lebanon, Yemen, Palestine and UAE) which have a high tobacco use prevalence compared with other reports. Future studies should use available resources to shift from repeatedly addressing the prevalence of smoking behaviors, attitudes among universities students in the Arab countries to focus on intervention and prevention strategies. They must monitor the prevalence of smoking through the surveillance system and records university students’ smoking behaviors. Future studies should focus on the psychosocial and economic determinants, as a means of finding strategies that encourage smoking cessation and prevention among universities students in the Arab countries.

**Table 1:** Prevalence of Smoking among Universities Students, Reported from Some Arab Countries

Country	Study Design	Participants No	Response Rate (%) ^a^	Males No	Females No	Age Range©	Smokers (%)	Males (%)	Females (%)	Cigarette Smokers (%)	Waterpipe Smokers (%)	Year ^b^	Authors
Yemen	Cross-sectional study	N=420	83.7	259	161	18-24 years	33.1	36.3	28.0	13.6	9.3	2019	Nasser and Zhang,
Yemen	Cross-sectional study	N=380	95	234	146	18-24 years	12.4	10.3	15.7	7.4	5.0	2018	Nasser et al.,
KSA	Cross-sectional study	N=23,424	99	23,424		18-25 years	30.4	30.4		70.7		2016	Ghaneem and Al-Nefisah,
Kuwait	Cross-sectional study		N=525*	525		16-32 years	46	46		13	10	2016	Husain et al.,
Bahrain	Cross sectional study	N=443	82.8	129	314	19≥22 years	10.8	27.0	4.2	24.9	2.0	2015	Alzayani and Hamadeh,
KSA	Cross-sectional		N=156*	156		18-22 years	42.3	42.3		34.8	21.2	2014	Hassan et al.,
Jordan	Cross-sectional study	N=547	70.1	151	396	19≥22 years	23.0	54.3	11.1	3.5	12.6	2014	Obeidat et al.,
Lebanon	Cross-sectional study	N=191	54.3	106	85	18-25 years	26.3			26.3	29.5	2013	Jradi et al.,
Tunisia	Cross-sectional survey	N=1123	93	346	777	18-28 years	14	38.4	3.4			2013	Maatouk et al.,
Egypt	Cross-sectional study	N=201	91.4	102	99	20-27 years	46.7	61.19	18.90	17.4	17.6	2012	Khan et al.,
Palestine	Cross-sectional study	N=954	95.4	480	474	18-20 years	34.7	52.7	16.5			2012	Musmar,
Syria	Cross-sectional study	N=583	75.3	394	189	17-28 years	20.8	26.1	9.5			2012	Al-Kubaisy et al.,
Libya	Cross-sectional study	N=304	95		304	19-25 years	28.3			80.2	19.8	2012	Abou-Faddan and Ahmed
KSA	Cross-sectional study	N=6686	90	2973	3713	17-25 years	14.5	32.7	5.9	48.2	36.4	2011	Mandil et al.,
KSA	Cross-sectional study	N=6686	90	2973	3713	≥ 15 years	14.5	32.7	5.9	48.1	36.3	2010	Mandil et al.,
Jordan	Cross-sectional study	N=712	88	369	343	17-28 years	35.0	56.9	11.4	80.0	19.3	2008	Khader and Alsadi,
Egypt	Cross-sectional study	N=600	94	263	337	17-25 years	7.7	17.5	0.0			2007	abolfotouh
UAE	Cross-sectional	N=1057	82	415	642	17-37 years	15.1	33.0	3.9	9.4	5.6	2007	Mandil et al.,
Jordan	Cross-sectional study	N=314	83.7	151	163	18-25 years	17.2	31.0	4.3			2006	Alomari et al.,

*Total number of respondents listed, as the response rate was not provided; a Response rate of the study; b Publication year; © Ages have been determined according to the educational systems and regulations of enrolment for the given countries
